# The autotaxin-LPA axis promotes membrane trafficking and secretion in yolk sac visceral endoderm cells

**DOI:** 10.1242/bio.060081

**Published:** 2023-10-30

**Authors:** Seiichi Koike, Kazuko Keino-Masu, Yoko Tanimoto, Satoru Takahashi, Masayuki Masu

**Affiliations:** ^1^ Graduate School of Comprehensive Human Sciences, University of Tsukuba, 1-1-1 Tennodai, Tsukuba, Ibaraki 305-8575, Japan; ^2^ Department of Molecular Neurobiology, Institute of Medicine, University of Tsukuba, 1-1-1 Tennodai, Tsukuba, Ibaraki 305-8575, Japan; ^3^ Laboratory of Organelle Synthetic Biology, Graduate School of Science and Engineering for Research, University of Toyama, 3190 Gofuku, Toyama-shi, Toyama 930-855, Japan; ^4^ Laboratory Animal Resource Center and Transborder Medical Research Center, University of Tsukuba, 1-1-1 Tennodai, Tsukuba, Ibaraki 305-8575, Japan

**Keywords:** Autotaxin, Endocytosis, Secretion, Mouse, Visceral endoderm, Yolk sac

## Abstract

Autotaxin, encoded by the *Enpp2* gene, is an exoenzyme that produces lysophosphatidic acid, thereby regulating many biologic functions. We previously reported that *Enpp2* mRNA was abundantly expressed in yolk sac visceral endoderm (VE) cells and that *Enpp2*^−/−^ mice were lethal at embryonic day 9.5 owing to angiogenic defects in the yolk sac. *Enpp2*^−/−^ mice showed lysosome fragmentation in VE cells and embryonic abnormalities including allantois malformation, neural tube defects, no axial turning, and head cavity formation. However, whether the defects in endocytic vesicle formation affect membrane trafficking in VE cells remained to be directly examined. In this study, we found that pinocytosis, transcytosis, and secretion of angiogenic factors such as vascular endothelial growth factor and transforming growth factor β1 were impaired in *Enpp2*^−/−^ VE cells. Moreover, pharmacologic inhibition of membrane trafficking phenocopied the defects of *Enpp2*^−/−^ mice. These findings demonstrate that *Enpp2* promotes endocytosis and secretion of angiogenic factors in VE cells, thereby regulating angiogenesis/vasculogenesis and embryonic development.

## Teaser

**Summary:** Autotaxin, encoded by the *Enpp2* gene, is required for pinocytosis, transcytosis, and secretion of angiogenic factors in murine yolk sac visceral endoderm cells, thereby regulating angiogenesis/vasculogenesis and embryonic development.

## INTRODUCTION

Autotaxin, also known as ectonucleotide pyrophosphatase/phosphodiesterase 2 (Enpp2), is a secreted lysophospholipase D that hydrolyzes extracellular lysophosphatidylcholine into lysophosphatidic acid (LPA; Tokumura et al., 2002; Umezu-Goto et al., 2002; Stefan et al., 2005; Perrakis and Moolenaar, 2014; Kano et al., 2022). LPA is a lipid mediator possessing a wide variety of biologic functions, including cell proliferation, migration, cytoskeletal modification, and survival (Luquain et al., 2003; Moolenaar et al., 2004). LPA activates 6 G protein-coupled receptors, LPA_1–6_, which induce intracellular signals via distinct G proteins (Yung et al., 2014; Kano et al., 2022). During early development [embryonic day 7.5 (E7.5)–E9.5], *Enpp2* mRNA was abundantly expressed in yolk sac visceral endoderm (VE) cells (Tanaka et al., 2006; Koike et al., 2009). *Enpp2*^−/−^ mice died at E9.5 owing to angiogenic defects in the yolk sac (Tanaka et al., 2006; van Meeteren et al., 2006; Koike et al., 2009). *Enpp2*^−/−^ embryos also showed allantois malformation, neural tube defects, no axial turning, and head cavity formation, indicating the essential roles of *Enpp2* for mouse embryonic development (Tanaka et al., 2006; van Meeteren et al., 2006; Koike et al., 2009, 2010).

The extraembryonic visceral yolk sac is composed of VE cells and the underlying mesoderm layer. Before the establishment of a chorioallantoic placenta (∼E9), VE cells function as an ‘early-placenta’ in the maternofetal exchange of nutrients. They endocytose maternal proteins, hydrolyze the proteins in lysosomes, and supply the digested products to the embryo proper as nutrients (Jollie, 1990; Bielinska et al., 1999; Zohn and Sarkar, 2010). Reflecting high endocytic and digestive activity, VE cells have large lysosomes, known as amorphous vacuoles in electron microscopic observation (Jollie, 1990; Bielinska et al., 1999; Koike et al., 2009). Inhibition of the membrane trafficking by use of chemicals, antibodies, or gene disruption caused fetal malformation (Lloyd, 1990; Bielinska et al., 1999), suggesting that membrane trafficking functions in VE cells are necessary for embryonic development. Moreover, VE cells secrete growth and angiogenic factors, thereby inducing vasculogenesis, angiogenesis, and hematopoiesis in the yolk sac (Belaoussoff et al., 1998; Goldie et al., 2008).

We previously revealed that lysosomes in VE cells were fragmented in *Enpp2*^−/−^mouse embryos (Koike et al., 2009). Moreover, we showed that LPA receptors (LPARs) and their downstream pathway, including Rho, Rho-associated coiled-coil containing protein kinase (ROCK), and LIM kinase (LIMK), were required to form large lysosomes. We also found that actin polymerization in VE cells was significantly reduced in *Enpp2*^−/−^ embryos and that pharmacologic perturbations of actin turnover dynamics in wild-type (WT) embryos resulted in the defects in lysosome formation (Koike et al., 2009). Because LIMK regulates actin dynamics (Bernard, 2007), we concluded that the autotaxin-LPAR-Rho-ROCK-LIMK pathway regulates lysosome formation via actin dynamics. Although the molecular mechanisms underlying the lysosome defects in *Enpp2*^−/−^ mice were clarified, whether malformation of the embryo proper is a consequence of dysfunction of VE cells or the direct result of *Enpp2* disruption in the embryo proper remained unknown.

In this study, we show that pinocytosis, transcytosis, and secretion of angiogenic factors such as vascular endothelial growth factor (VEGF) and transforming growth factor β1 (TGF-β1) are impaired in *Enpp2*^−/−^ VE cells. Furthermore, we demonstrate that pharmacologic perturbation of membrane trafficking phenocopied the defects in *Enpp2*^−/−^ mice. These findings demonstrate that *Enpp2* promotes membrane trafficking in VE cells, thereby regulating angiogenesis/vasculogenesis and embryonic development.

## RESULTS

### Autotaxin enhances endocytosis

We previously showed that in *Enpp2*^−/−^ VE cells, lysosomes were smaller and accumulation of maternal IgG in the lysosomes was reduced (Koike et al., 2009). These phenotypes imply defects in endocytic trafficking in *Enpp2*^−/−^ VE cells. However, the membrane trafficking functions of VE cells remained to be directly examined.

**Fig. 1.**
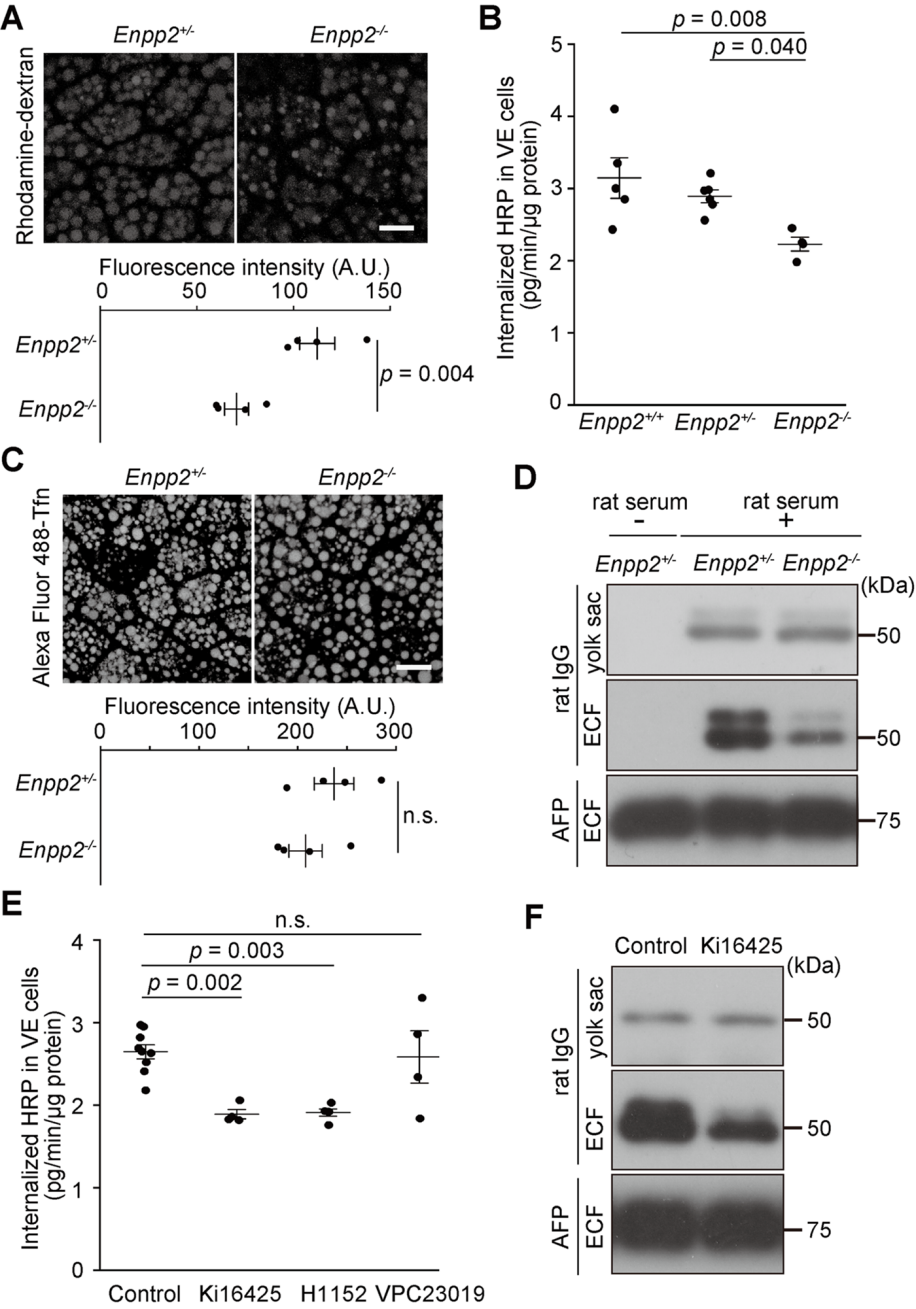
***Enpp2* knockout impairs pinocytosis and transcytosis in VE cells.** (A) Pinocytosis in E8.5 VE cells. After incubation with 100 µg/ml rhodamine B-labeled dextran at 37°C for 3 min, confocal images of VE cells were obtained. Scale bar: 10 µm. The fluorescence intensity of rhodamine B in VE cells (arbitrary units, A.U.) was lower in *Enpp2*^−/−^ embryos (*n*=4, unpaired *t*-test). (B) HRP uptake by E8.5 VE cells. After embryos were incubated with 100 µg/ml HRP at 37°C for 3 min, HRP activity in the dissected yolk sac was measured. HRP uptake was significantly lower in the *Enpp2*^−/−^ embryos than in the *Enpp2*^+/+^ and *Enpp2*^+/−^ embryos (*n*=5 for *Enpp2*^+/+^, *n*=6 for *Enpp2*^+/−^, *n*=4 for *Enpp2*^−/−^; one-way ANOVA with the Tukey multiple comparison test). (C) Receptor-mediated endocytosis in E8.5 VE cells. After incubation with 20 µg/ml Alexa488-transferrin (Tfn) at 37°C for 3 min, confocal images of VE cells were obtained. Scale bar: 10 µm. The fluorescence intensity of Alexa488-transferrin in VE cells did not differ between the control and the *Enpp2*^−/−^ embryos (*n*=4, unpaired *t*-test). (D) Transcytosis across VE cells. After E7.5 whole embryos were cultured in 100% rat serum overnight, rat IgG in the yolk sacs and ECF was detected by use of Western blot analysis. Approximately equal amounts of rat IgG were detected in the yolk sacs of the control and the *Enpp2*^−/−^ embryos, whereas the amount of rat IgG in the ECF was much lower in the *Enpp2*^−/−^ embryos than in the control. No rat IgG was detected in the ECF of E8.5 *Enpp2*^+/−^ embryos without culture (left column), indicating that the above signals in the ECF were derived from transcytosed rat IgG across the VE cells. AFP protein was used as the internal control. (E) HRP uptake after treatment with endocytic inhibitors. E7.5 WT embryos were cultured with an LPA_1_/LPA_3_ antagonist (Ki16425, 10 µM), an S1P_1_/S1P_3_ antagonist (VPC23019, 10 µM), or a ROCK inhibitor (H1152, 0.1 µM) for 1 d. The next day, after the embryos were incubated in 100 µg/ml HRP at 37°C for 3 min, HRP activity in the dissected yolk sac was measured. HRP uptake was significantly reduced by treatment with Ki16425 and H1152 but not by treatment with VPC23019 (*n*=9 for the control, *n*=4 for Ki16425, *n*=5 for H1152, and *n*=4 for VPC23019; one-way ANOVA with the Tukey multiple comparison test). (*F*) Transcytosis reduced by treatment with Ki16425. Culture of E7.5 embryos in the presence of 10 µM Ki16425 for 1 d reduced transcytosis of rat IgG across the VE cells. Abbreviation: n.s., not significant.

Therefore, we investigated the endocytic activity of VE cells. In general, extracellular materials are internalized by means of several different pathways, including pinocytosis and receptor-mediated endocytosis (Doherty and McMahon, 2009). First, to quantify pinocytosis, in which small molecules are internalized with bulk fluid, E8.5 whole embryos were incubated with 100 µg/ml rhodamine B-labeled dextran at 37°C for 3 min. Because VE cells are localized at the outermost layer of embryos, VE cells take up dextran from the culture medium, and the amount of internalized dextran can be quantified by means of fluorescence microscopy. In a pilot experiment, we confirmed that a short pulse time (3 min) was sufficient to observe the fluorescence of the internalized marker, most likely owing to the high endocytic activity of VE cells. When the fluorescence intensities of the internalized dextran in VE cells were quantitated as a measure of endocytosis, the signals were significantly lower in the *Enpp2*^−/−^ embryos than in the control (Fig. 1A). We then examined another pinocytosis marker, horse radish peroxidase (HRP). After whole E8.5 embryos were incubated with 100 µg/ml HRP at 37°C for 3 min, the enzyme activity in VE cells was measured. This analysis revealed that HRP uptake was also significantly lower in the *Enpp2*^−/−^ embryos than in the WT and heterozygous embryos (Fig. 1B). Taken together, these data indicate that pinocytosis is impaired in *Enpp2*^−/−^ VE cells.

Next, we examined receptor-mediated endocytosis, in which ligand molecules are bound with specific receptors on the cell surface and internalized with their receptors. For this purpose, we first used transferrin, a well-studied marker for receptor-mediated endocytosis. After whole embryos were incubated with 20 µg/ml Alexa Fluor 488-conjugated transferrin at 37°C for 3 min, the fluorescence intensity in VE cells was measured. The amounts of endocytosed transferrin did not differ between the control and the *Enpp2*^−/−^ embryos (Fig. 1C). We then examined IgG, another marker for receptor-mediated endocytosis, which is internalized via the neonatal Fc receptor (FcRn) on VE cells from maternal tissues (Kim et al., 2009). We cultured whole E7.5 mouse embryos in rat serum *ex vivo* for 16 h and examined endocytosed rat IgG in the yolk sac by use of Western blot analysis. The yolk sac of noncultured E8.5 embryos did not show any signal detected by anti-rat IgG antibody, whereas the signals were detected in the yolk sac after it had been cultured in rat serum (Fig. 1D), suggesting that the antibody detected endocytosed rat IgG specifically and did not react with mouse IgG. The amount of rat IgG in the yolk sac did not differ between the control and the *Enpp2*^−/−^ VE cells (Fig. 1D, Fig. S1A). Moreover, the same levels of rat IgG were detected in the control and the *Enpp2*^−/−^ VE cells when culture in rat serum was shortened to 3 h (data not shown). These data indicate that receptor-mediated endocytosis was hardly affected in *Enpp2*^−/−^ VE cells. However, the levels of rat IgG in the extraembryonic coelomic fluid (ECF) were much lower in the *Enpp2*^−/−^ VE cells than in the control, whereas the levels of alpha-fetoprotein (AFP), the most abundant protein in the yolk sac, in the ECF were approximately the same in the control and the *Enpp2*^−/−^ embryos (Fig. 1D, Fig. S1A). These results suggest that transcytosis from the maternal side to the embryonic side was impaired in *Enpp2*^−/−^ VE cells.

### The autotaxin-LPA-LPAR axis regulates pinocytosis and transcytosis

Previously, we reported that *Enpp2* was required for the formation of large lysosomes in VE cells and that the molecular mechanisms underlying the phenotype included LPARs, the Rho-ROCK-LIMK pathway, and actin turnover dynamics (Koike et al., 2009). We thus examined whether this signaling pathway was also required for pinocytosis and transcytosis in VE cells. When E7.5 WT embryos were cultured in the presence of an LPA_1_/LPA_3_ antagonist (10 µM Ki16425) or a ROCK inhibitor (0.1 µM H1152), HRP pinocytosis was significantly reduced (Fig. 1E), but an S1P_1_/S1P_3_ antagonist (10 µM VPC23019) did not affect HRP pinocytosis (Fig. 1E). In addition, Ki16425 also reduced transcytosis (Fig. 1F, Fig. S1B), similar to the reduction observed in *Enpp2*^−/−^ VE cells. These findings indicate that the LPAR–ROCK pathway regulates pinocytosis and transcytosis downstream of autotaxin.

### Receptor degradation is impaired in *Enpp2*^−/−^ VE cells

**Fig. 2.**
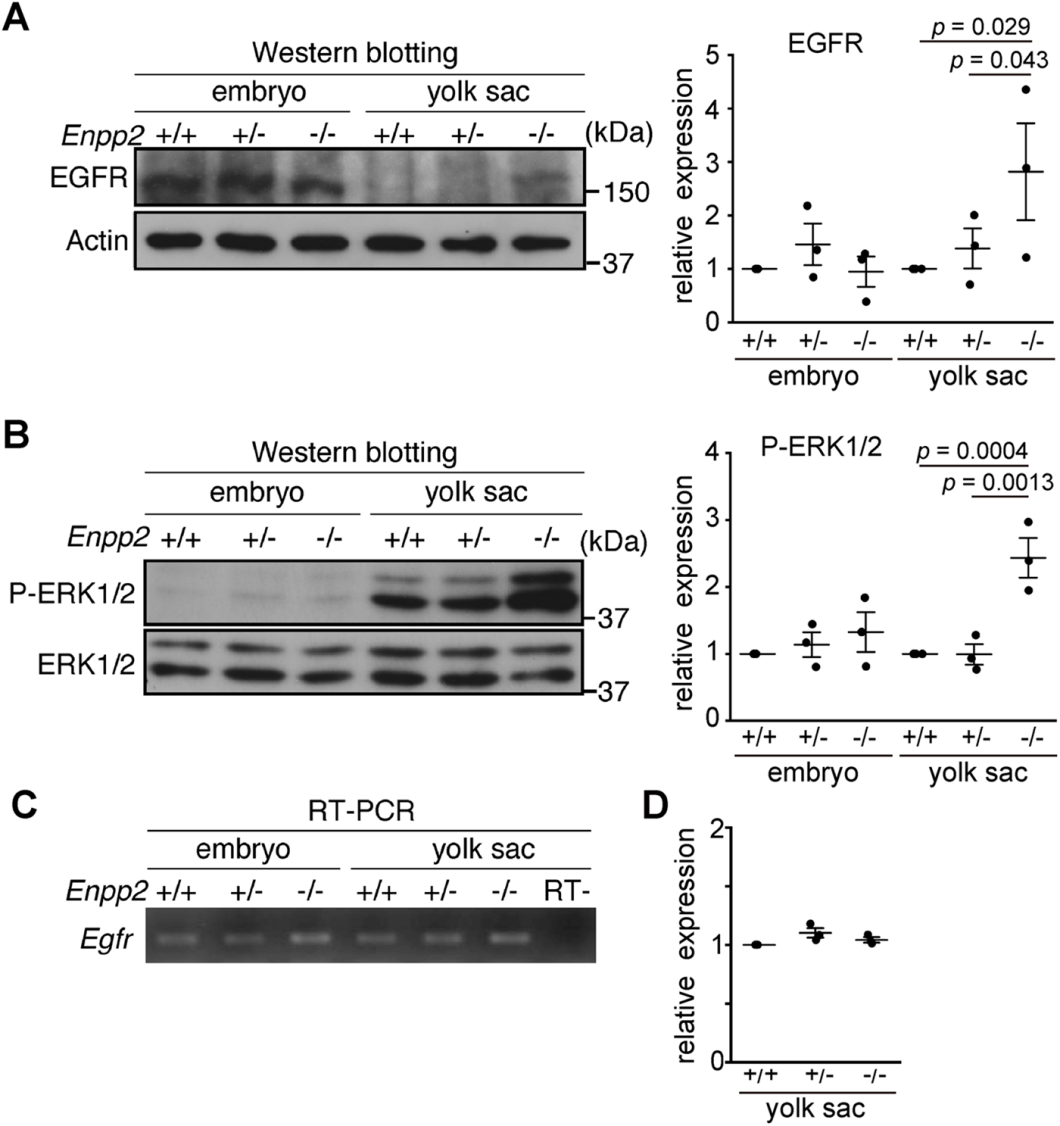
***Enpp2* knockout impairs EGFR degradation in the yolk sac.** (A) Representative Western blot of EGFR in the embryo and yolk sac at E8.5 (left). The relative expression of EGFR protein, normalized to β-actin expression, is shown with the control *Enpp2*^+/+^ as 1 (right; *n*=3, unpaired *t*-test). (B) Representative Western blot of phospho-ERK1/2 (P-ERK1/2), and total ERK1/2 in the embryo and yolk sac at E8.5 (left). The relative expression of phospho-ERK1/2 protein, normalized to EPK1/2 expression, is shown with the control *Enpp2*^+/+^ as 1 (right; *n*=3, unpaired *t*-test). (C) RT-PCR analysis of *Egfr* in the embryo and yolk sac at E8.5. RT- indicates a negative control in which a reverse transcriptase was omitted from the reaction. (D) Real-time PCR analysis of *Egfr* RNA in the yolk sac at E8.5. The relative expression of *Egfr*, normalized to *Actb* expression, is shown with the control *Enpp2*^+/+^ as 1 (*n*=3).

In general, endocytosed materials are recycled back to the plasma membrane or sorted to late endosomes and degraded after fusion with lysosomes. Epidermal growth factor receptor (EGFR) is activated and phosphorylated by ligand binding, followed by endocytosis and degradation in lysosomes for terminating EGFR signaling (Avraham and Yarden, 2011). To evaluate the trafficking of endocytosed receptors to lysosomes, we examined EGFR degradation in *Enpp2*^−/−^ VE cells. Western blot analysis showed that *Enpp2* disruption increased EGFR protein levels in the yolk sac but not in the embryo proper (Fig. 2A). Furthermore, extracellular signal-regulated kinase (ERK), a downstream molecule of the EGF signaling pathway, was robustly phosphorylated in the *Enpp2*^−/−^ yolk sac, whereas the level of total ERK1/2 was not changed or else was slightly reduced (Fig. 2B). Given that the *Egfr* mRNA expression level in the yolk sac was not changed (Fig. 2C,D), these data suggest that the degradation of the EGF–EGFR complex was reduced, leading to the overactivation of the EGFR–ERK pathway in the *Enpp2*^−/−^ yolk sac.

### Protein secretion from VE cells is impaired in *Ennp2*^−/−^ embryos

**Fig. 3.**
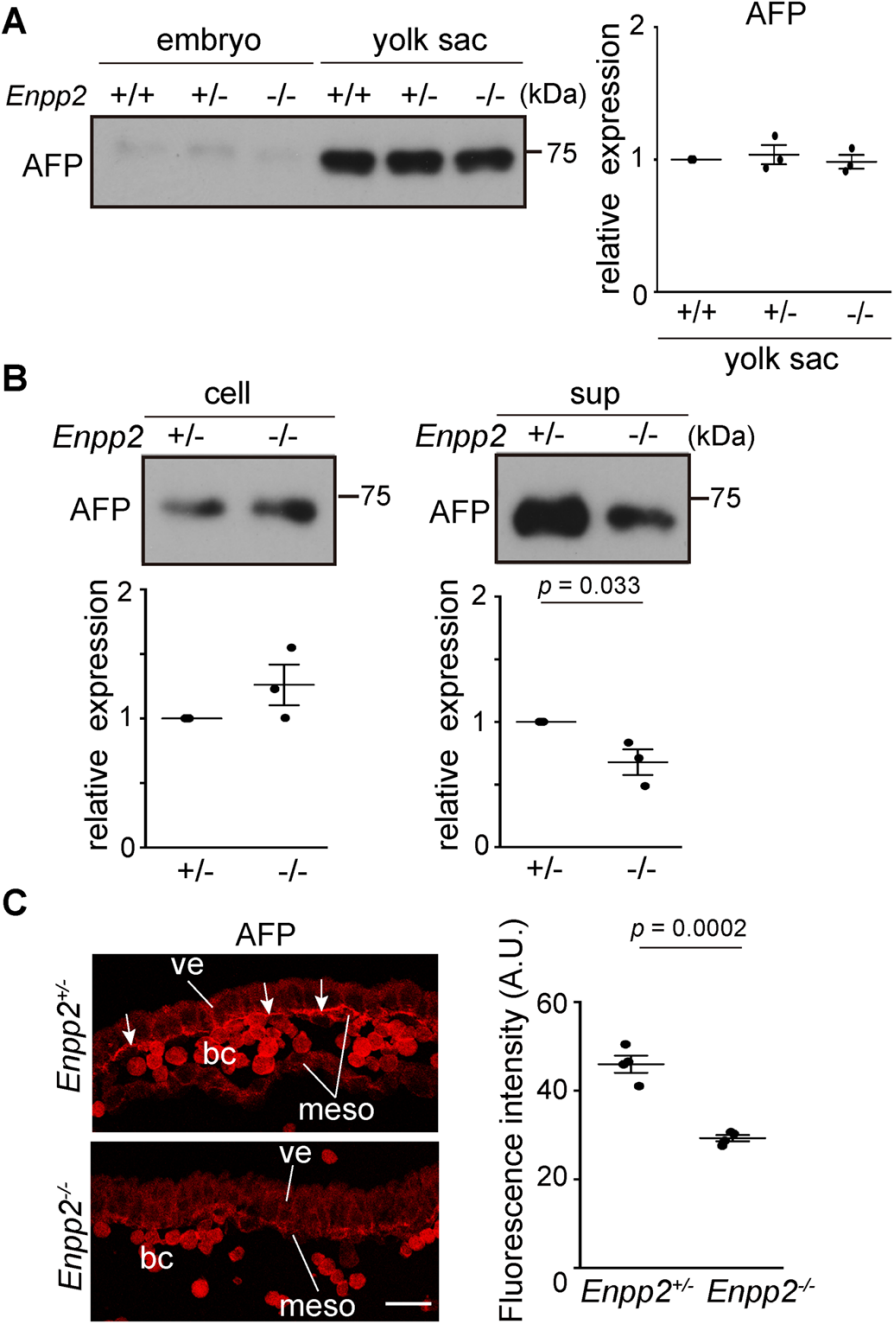
***Enpp2* knockout impairs protein secretion from VE cells.** (A) Representative Western blot of AFP in the embryos and yolk sacs at E8.5. The relative expression of AFP in the yolk sac at E8.5 is shown with the control *Enpp2*^+/+^ as 1 (right; *n*=3, unpaired *t*-test). (B) Western blot analysis of AFP in the cell lysates (cell) and culture supernatants (sup). After the yolk sacs of the E8.5 embryos were dissected and cultured *in vitro* at 37°C for 6 h, the cell lysates and supernatants were subjected to Western blot analysis. The relative expression of AFP protein level is shown with the control *Enpp2*^+/−^ as 1 (lower; *n*=3, unpaired *t*-test). AFP was decreased in the supernatant, but not in the yolk sac cells of the *Enpp2*^−/−^ embryos. (C) Immunostaining of AFP in the yolk sac region of the E8.5 embryos. Cell surface localization of AFP was examined by use of unpermeabilized yolk sacs with anti-AFP antibody and subsequent sectioning. AFP was detected on the basal surface of the VE cells (ve) in the *Enpp2*^+/−^ embryos (white arrows), but not in the *Enpp2*^−/−^ embryos. The relative fluorescence intensities at the basal surface of the VE cells are shown (right; *n*=4, unpaired *t*-test). Abbreviations: bc, blood cells; meso, mesoderm cells. Scale bar: 25 µm.

VE cells secrete many proteins to support embryonic growth. Inhibition of nutrient transport of VE cells by chemicals (eg, Trypan blue) or antibodies against the visceral yolk sac resulted in teratogenic effects (Brent et al., 1990; Lloyd, 1990). Given that *Afp* mRNA is specifically expressed in VE cells at early embryonic stages and AFP protein is secreted into the ECF (Kwon et al., 2006), we examined the secretory function of VE cells by analyzing AFP. Western blot analysis showed that AFP was equally expressed in the control and in the *Enpp2*^−/−^ yolk sac (Fig. 3A). To directly quantify protein secretion from the yolk sac, the yolk sacs of E8.5 embryos were dissected and individually cultured for 6 h in a culture medium. The amounts of AFP protein secreted into the medium were significantly lower in the *Enpp2*^−/−^ embryos, whereas those in the yolk sac cells did not differ between the control and the *Enpp2*^−/−^ embryos (Fig. 3B). In addition, immunostaining of AFP using unpermeabilized yolk sacs detected the cell surface localization of AFP on the basal side of the VE cells of the E8.5 WT embryos, whereas such extracellular AFP localization was hardly detectable in the *Enpp2*^−/−^ embryos (Fig. 3C). These data indicate that the yolk sac VE cells produced AFP normally, but AFP secretion from VE cells was impaired in the *Enpp2*^−/−^ embryos, suggesting that autotaxin is required for protein secretion from VE cells.

### Secretion of angiogenic factors is impaired in *Enpp2*^−/−^ VE cells

**Fig. 4.**
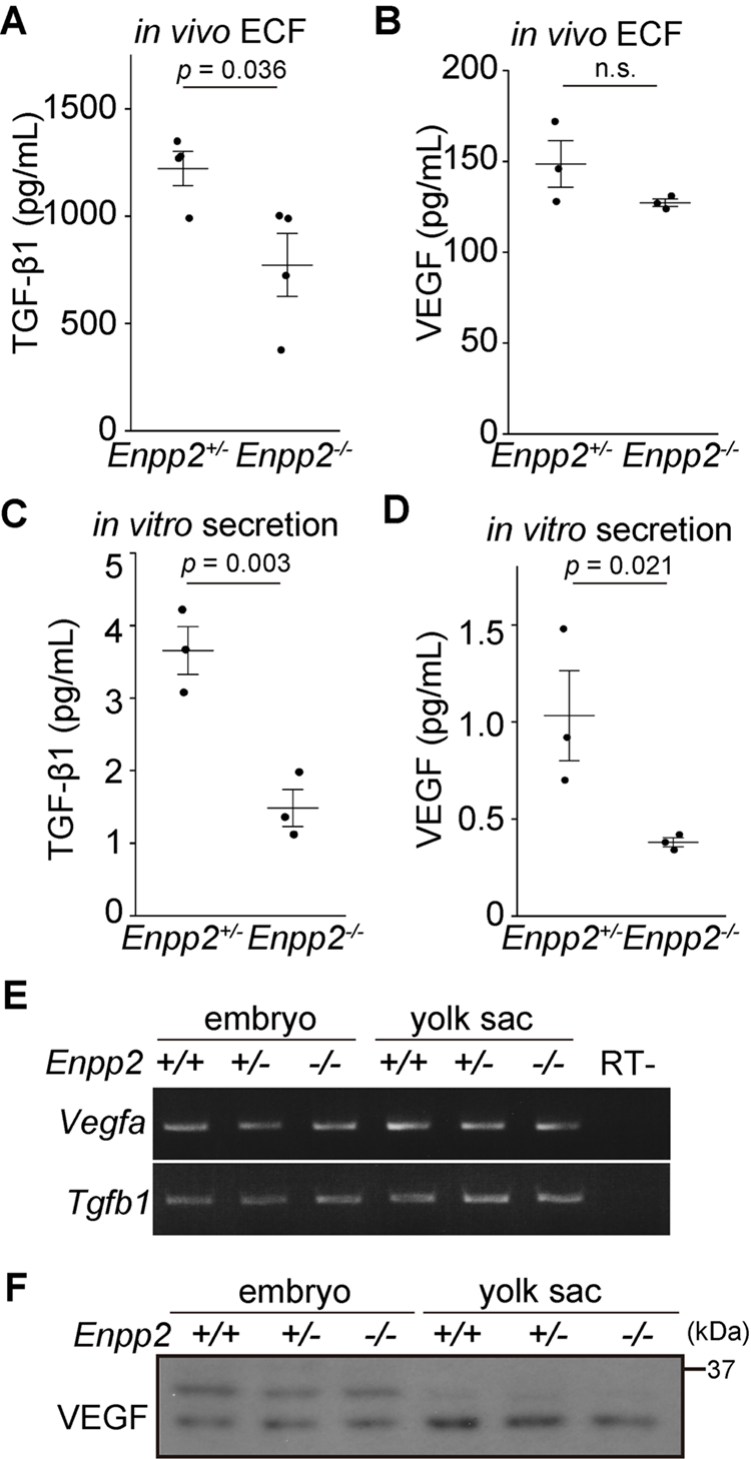
***Enpp2* knockout impairs secretion of angiogenic factors from the yolk sac.** (A-B) Concentration of TGF-β1 (A) and VEGF (B) in the ECF of *Enpp2*^+/−^ and *Enpp2*^−/−^ embryos at E8.5 (*n*=4 in [A], *n*=3 in [B], unpaired *t*-test). (C-D) Amount of TGF-β1 and VEGF secreted from the yolk sac. After the yolk sac was incubated for 16 h at 37°C in a culture medium, TGF-β1 and VEGF concentrations in the medium were measured by means of ELISA. Secretion of TGF-β1 and VEGF was reduced in the *Enpp2*^−/−^ yolk sacs (*n*=3, unpaired *t*-test). (E) RT-PCR of *Vegfa* and *Tgfb1* in the embryo and yolk sac of *Enpp2*^+/+^, *Enpp2*^+/−^, and *Enpp2*^−/−^ at E8.5. RT- indicates a negative control in which a reverse-transcriptase was omitted from the reaction. (F) Western blot analysis of VEGF in the embryo and yolk sac of E8.5 mice. Abbreviation: n.s., not significant.

VE cells also secrete angiogenic factors, such as VEGF, TGF-β1, and FGF2, to support vasculogenesis/angiogenesis and hematopoiesis in the yolk sac (Belaoussoff et al., 1998; Goldie et al., 2008). *Vegfa*-knockout mice die at E7.5 owing to angiogenic defects (Carmeliet et al., 1996; Ferrara et al., 1996). However, blood island formation and vascular development in the yolk sac of *Vegfa*-knockout mice were rescued by introduction of WT cells into the visceral endoderm, suggesting that VEGFA secreted from VE cells is sufficient for angiogenesis in the yolk sac (Damert et al., 2002). We wondered whether defects in angiogenic factor secretion from *Enpp2*^−/−^ VE cells cause blood vessel abnormalities in the yolk sac. To address this question, we first measured the concentrations of TGF-β1 and VEGF in the ECF *in vivo* by use of ELISA. In the *Enpp2*^−/−^ ECF, the concentration of TGF-β1 was significantly lower than that in the control, whereas the concentration of VEGF was slightly decreased but the difference did not reach significance (Fig. 4A,B). To test the secretion activity of VE cells, the yolk sac was isolated and incubated in a culture medium at 37°C for 16 h, and the concentrations of TGF-β1 and VEGF in the medium were measured. The concentrations of both TGF-β1 and VEGF in the medium were significantly lower in the *Enpp2*^−/−^ yolk sac (Fig. 4C,D). Given that *Vegfa* and *Tgfb1* mRNA expression (Fig. 4E, Fig. S1C-D) and the VEGF protein level (Fig. 4F, Fig. S1E) in the yolk sac did not differ between the control and the *Enpp2*^−/−^ yolk sac, these data indicate that the secretions of VEGF and TGF-β1 were reduced in the *Enpp2*^−/−^ yolk sac, although we could not examine TGF-β1 protein levels because Western blot analysis using the antibodies tested could not detect TGF-β1. Taken together, the results indicate that autotaxin is indispensable for secretion of angiogenic factors from VE cells.

### Introduction of WT cells into the visceral endoderm restores the phenotypes of *Ennp2*^−/−^ embryos

Previous studies revealed that *Enpp2*^−/−^ embryos showed not only lysosomal defects in VE cells but also developmental abnormalities in the embryo proper (Tanaka et al., 2006; van Meeteren et al., 2006; Koike et al., 2009, 2010). However, whether the phenotypes in the *Enpp2*^−/−^ embryo proper were caused by the dysfunction of VE cells or by the absence of the *Enpp2* gene in the embryo proper remained unknown. To distinguish between these two possibilities, we performed a tetraploid complementation experiment. Because the descendants of tetraploid cells contribute to the extraembryonic tissues (VE cells and the trophoectoderm) but not to the embryo proper nor to the mesodermal cells in the yolk sac (Hadjantonakis et al., 1998; Tam and Rossant, 2003; Tanaka et al., 2009), in the tetraploid/diploid chimeric mice, the embryo proper contains only diploid cells, whereas VE cells contain both diploid and tetraploid cells. Therefore, it is possible to distinguish whether the defects are caused by embryonic or by extraembryonic effects of the mutation by analyzing the phenotypes in tetraploid/diploid chimeras (Tanaka et al., 2009).

We thus created chimeras by aggregating tetraploid WT embryos expressing enhanced green fluorescent protein (EGFP) and diploid EGFP-negative *Enpp2*^−/−^ embryos (Fig. S2A). Because we used diploid embryos obtained from *Enpp2* heterozygous intercrossing, we selected *Enpp2*^−/−^ embryos by genotyping EGFP-negative embryos proper at E9.5. Of the 44 embryos collected at E9.5, 13 embryos were chimeras in VE cells: among them, 1 embryo proper was negative for EGFP and *Enpp2*^−/−^; in the other 12 embryos, the embryos proper were composed of EGFP-positive cells and stopped normal development, suggesting that they were derived from tetraploid cells (Goto et al., 2002; Eakin et al., 2005). We thus examined an embryo having chimeric VE cells and an *Enpp2*^−/−^ embryo proper. As shown in Fig. S2B-G, the embryo appeared to be developing almost normally at E9.5, showing normal axial turning and no exencephaly (Fig. S2G). Given that the development of *Enpp2*^−/−^ embryos was arrested at E8.5 and that all of them were much smaller and did not undergo embryonic turning at E9.5 (Tanaka et al., 2006; van Meeteren et al., 2006; Koike et al., 2009), the *Enpp2*^−/−^ embryo obtained by the tetraploid/diploid chimera was assumed to undergo normal development. Although the observation of only one case is insufficient to draw a conclusion, these data suggest the possibility that the phenotypes in the *Enpp2*^−/−^ embryo proper are attributable to the defects in VE cells.

Next, we examined restoration of VE cell functions. As shown before, VE cells endocytose maternal IgG from the apical side facing the maternal tissue. Immunostaining of mouse endogenous IgG revealed strong signals in EGFP-negative *Enpp2*^−/−^ VE cells surrounded by WT VE cells in addition to EGFP-positive WT VE cells (Fig. S2H-J) but weak or no signals in the area composed of only EGFP-negative *Enpp2*^−/−^ VE cells (Fig. S2K-M). Similar results were observed in different areas of the yolk sac (data not shown). Quantification of the fluorescence intensity in these areas indicated that IgG internalization was significantly decreased in the area composed of EGFP-negative *Enpp2*^−/−^ VE cells (Fig. S2N). These data indicate that *Enpp2* expression in VE cells is required for active IgG endocytosis into VE cells and that autotaxin can act on neighboring cells in a paracrine manner. Furthermore, blood vessels, depicted by immunostaining of platelet endothelial cell adhesion molecule 1 (PECAM-1), formed normally in the region facing EGFP-positive WT VE cells (Fig. S2H-J), whereas abnormal dilatation of blood vessels, which was also seen in *Enpp2*^−/−^ yolk sacs (Koike et al., 2009), was observed in the region facing EGFP-negative *Enpp2*^−/−^ VE cells (Fig. S2K-M). These data suggest that *Enpp2* expression in VE cells is necessary for normal angiogenesis/vasculogenesis in the yolk sac.

### Endocytosis inhibitors lead to head-cavity formation

**Fig. 5.**
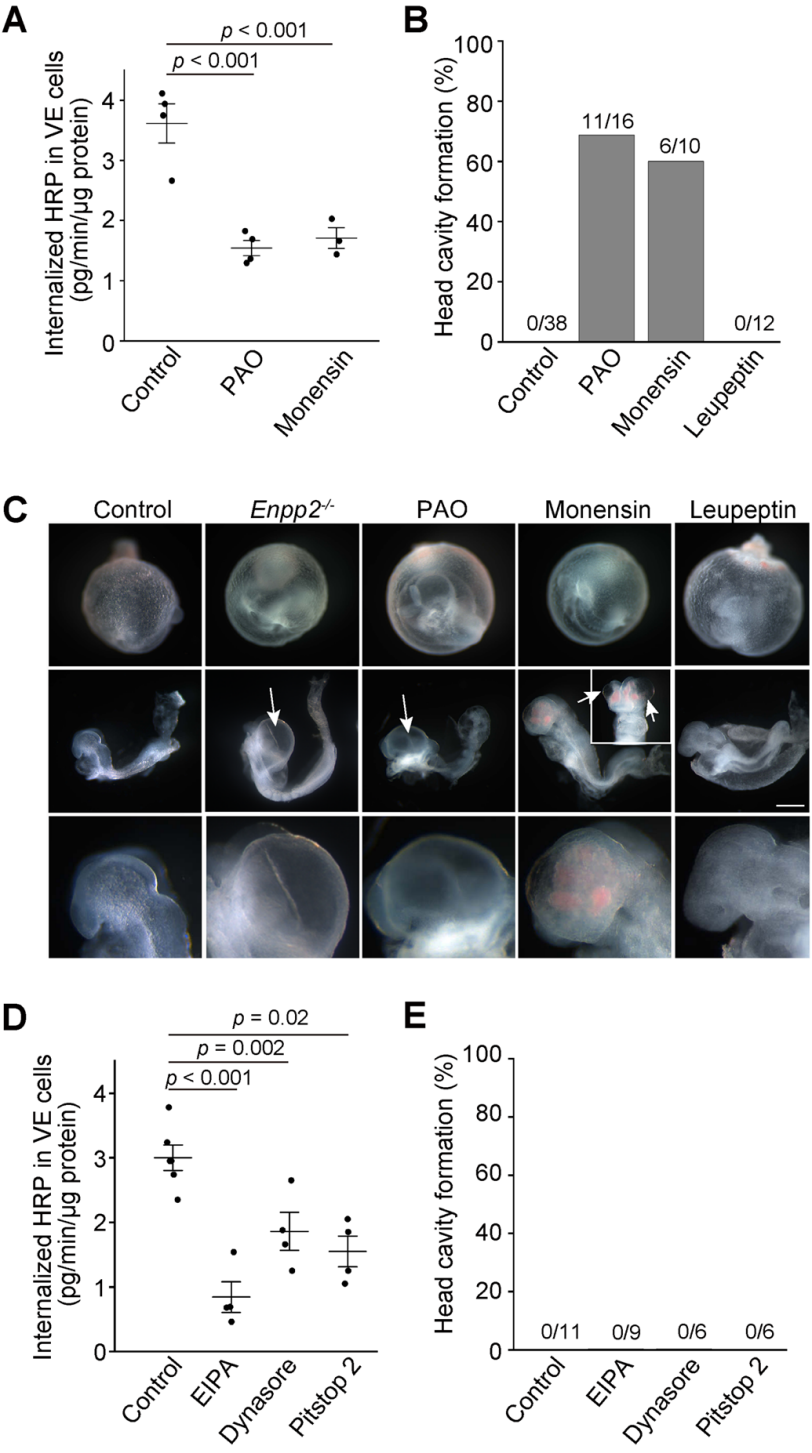
**Endocytosis inhibitors lead to head cavity formation.** (A) HRP uptake of VE cells after endocytosis inhibitor treatment. E7.5 WT embryos were cultured with an endocytosis inhibitor, 0.1 μM phenylarsine oxide (PAO) or 50 nM monensin, for 1 d. The next day, after the embryos were incubated with 100 µg/ml HRP at 37°C for 3 min, HRP activity in the dissected yolk sac was determined. HRP uptake was significantly reduced by the treatment with endocytosis inhibitors (*n*=4, one-way ANOVA with the Tukey multiple comparison test). (B,C) Head cavity formation in *Enpp2*^−/−^ embryos and in embryos treated with endocytosis inhibitors. PAO and monensin induced head cavities at rates of 69% (11/16) and 60% (6/10), respectively, whereas 25 µg/ml leupeptin, a lysosomal inhibitor, did not. The upper and middle panels in (C) show the appearance of the whole embryos and embryos proper after 1-day culture, respectively. The inset and arrows show the appearance of the head region of the embryo and head cavities, respectively. The lower panels show the magnified images of the head regions. Scale bars: 400 µm for upper and middle figures and 120 µm for lower figures. (D) HRP uptake of VE cells after endocytosis inhibitor treatment. After E7.5 WT embryos were cultured with an inhibitor for micropinocytosis (EIPA, 50 μM) or inhibitors for clathrin-dependent endocytosis (dynasore, 20 μM; pitstop 2, 10 μM) for 1 d, HRP uptake was measured. HRP uptake was significantly reduced by the treatment with all these inhibitors. (E) Head cavity formation after pharmacologic treatment. None of the embryos showed head cavity formation after treatment with EIPA, dynasore, or pitstop 2.

Head-cavity formation is one of the characteristic phenotypes observed in *Enpp2*^−/−^ embryos (Tanaka et al., 2006; van Meeteren et al., 2006; Koike et al., 2009, 2010). However, the molecular mechanisms underlying the cavity formation remain unknown. Previous studies revealed that a potent teratogen, Trypan blue, induced similar head cavities in rat embryos both *in vitro* and *in vivo* (Rogers et al., 1985) and that Trypan blue was mainly accumulated in the lysosomes of VE cells and inhibited lysosomal enzymes (Beck, et al., 1967). In addition, abnormal osmolarity of the ECF was observed after embryos were exposed to Trypan blue, suggesting that the transport defects of VE cells may result in the cavity formation (Rogers, et al., 1985). Thus, we wondered whether inhibition of endocytosis or lysosomal functions induces head-cavity formation. When E7.5 whole embryos were cultured with broad-spectrum inhibitors of membrane trafficking, 0.1 µM phenylarsine oxide (PAO) or 50 nM monensin, for 1 d, HRP pinocytosis was greatly reduced in VE cells (Fig. 5A). Under the same condition, head cavities were formed with high frequency, whereas inhibition of lysosomal functions by 25 µg/ml leupeptin did not induce head-cavity formation (Fig. 5B,C). When *Enpp2*^−/−^ embryos were cultured in the same condition, most of them had head cavities (Fig. 5C), at a frequency of 90.9% (Koike et al., 2010). Next, we tested more specific inhibitors for endocytosis processes. Treatment of embryos with an inhibitor for micropinocytosis, 5-(*N*-ethyl-*N*-isopropyl)-amiloride (EIPA, 50 µM), or inhibitors for clathrin-dependent endocytosis, dynasore (20 µM) or pitstop 2 (10 µM), reduced HRP internalization significantly in VE cells (Fig. 5D). As expected, EIPA had the strongest effects on HRP uptake when compared with the inhibitors of clathrin-dependent endocytosis (Fig. 5D). However, none of these inhibitors induced head-cavity formation (Fig. 5E).

## DISCUSSION

**Fig. 6.**
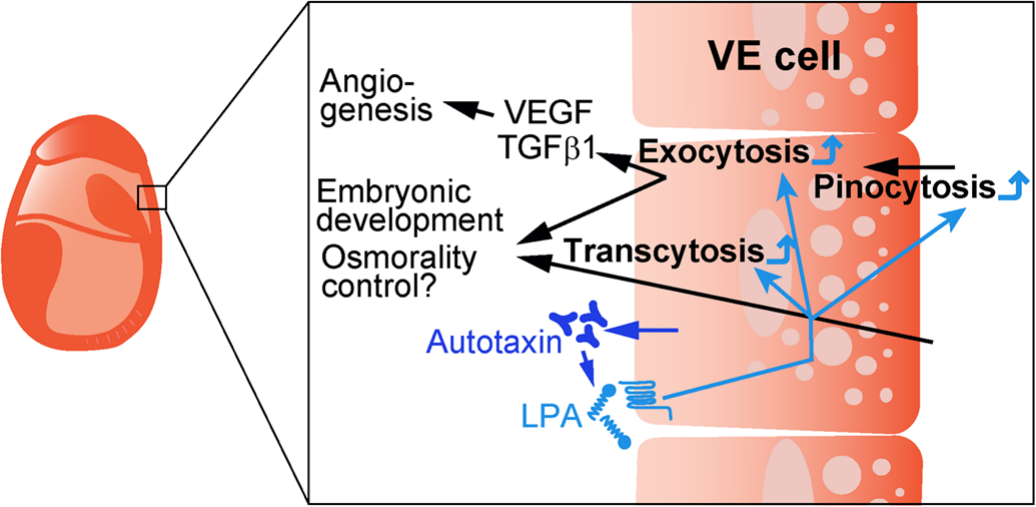
**Autotaxin functions in endocytosis and protein secretion in VE cells.** LPA generated by autotaxin activates membrane trafficking including in pinocytosis, transcytosis, and exocytosis in yolk sac VE cells of mouse embryos.

In this study, we investigated the roles of autotaxin in membrane trafficking in yolk sac VE cells and embryonic development. We directly demonstrated that pinocytosis, receptor degradation after endocytosis, transcytosis, and secretion of proteins were significantly decreased in *Enpp2*^−/−^ VE cells. Given that VE cells endocytose ions, proteins, and amino acids from the maternal side and secrete them into the ECF (Jollie,1990; Bielinska et al., 1999), which are essential for embryonic growth, our data indicate that the functional impairment of VE cells leads to abnormalities in *Enpp2*^−/−^ embryos, most likely owing to defects in membrane trafficking in VE cells (Fig. 6).

Secretion of AFP and VEGF into the culture medium was significantly decreased in *Enpp2*^−/−^ yolk sac when compared with in the control (Figs 3B and 4D), although the concentrations of AFP and VEGF proteins in the ECF did not differ between the control and the *Enpp2*^−/−^ embryos (Figs 1D and 4B). This discrepancy seems to arise from the differences in the *in vivo* and *in vitro* environments: the *in vitro* data simply reflect the secretion of these factors, whereas the *in vivo* data show their net amounts determined by a balance between secretion and clearance/degradation processes. TGF-β1 appears to be differently regulated from AFP and VEGF because the protein levels of TGF-β1 were lower in both the *in vivo* and the *in vitro* conditions (Fig. 4A,C).

Our pharmacologic experiments demonstrated that the formation of the head cavity, one of the typical phenotypes in *Enpp2*^−/−^ embryos, occurred as a result of treatment with PAO and monensin, but not with other endocytosis inhibitors, such as EIPA, dynasore, and pitstop 2 (Fig. 5). PAO, a sulfhydryl-modifying reagent, and monensin, an ionophore, have multiple cellular effects primarily targeting endocytosis, which includes both clathrin-dependent endocytosis and micropinocytosis (Gibson et al., 1989; Frost et al., 1989; Dickson et al., 1982; Wilcox et al., 1982), whereas EIPA specifically inhibits macropinocytosis, and dynasore and pitstop 2 are specific inhibitors of clathrin-dependent endocytosis. We hypothesize that head cavity formation occurs as a result of combined inhibition of multiple trafficking pathways rather than of inhibition of a single endocytosis pathway, because endocytosis, transcytosis, and secretion are impaired in *Enpp2*^−/−^ VE cells. Future investigations are necessary to determine which trafficking pathway plays a crucial role in the formation of the head cavity.

In the tetraploid complementation experiment, the endocytic defects were rescued in the small patches of *Enpp2*^−/−^ VE cells surrounded by WT VE cells but not in the *Enpp2*^−/−^ VE cells not surrounded by WT VE cells. These data indicate that autotaxin acts only at a limited distance in a paracrine manner although it is efficiently secreted (Koike et al., 2006). It was reported that LPA in the extracellular space is rapidly degraded by lipid phosphate phosphatases (Tang et al., 2015) and that binding of autotaxin to the cell surface helps to protect it from enzymatic degradation (Peyruchaud et al., 2019). The somatomedin B domain of autotaxin interacts with β3-integrins of activated platelets and lymphocytes, leading to enhancement of their motility (Kanda et al., 2008; Pamuklar et al., 2009; Fulkerson et al., 2011). Integrin activation likely serves as a mechanism to recruit autotaxin to the cell surface and deliver LPA to its cognate receptors. Furthermore, the interaction of autotaxin with integrins induces a conformational change of autotaxin to promote LPA release (Hausmann et al., 2011). Our observation that autotaxin has a spatially limited range of action provides the first *in vivo* evidence supporting the findings of the above previous reports.

We revealed that autotaxin, LPAR, and ROCK are required for endocytosis and transcytosis in VE cells. This is reminiscent of our previous findings that the LPAR-Rho-ROCK-LIM kinase pathway and actin polymerization are necessary for large lysosome formation in VE cells (Koike et al., 2009). Given that LPA signaling remodels actin dynamics (Mir et al., 2020) and that the actin cytoskeleton is involved in membrane trafficking (Lanzetti, 2007), the defects in intracellular membrane trafficking in *Enpp2*^−/−^ VE cells are likely related to and caused by dysregulation of the Rho-ROCK-LIM kinase pathway. Association between LPA and membrane trafficking was reported in other cell systems: LPA enhanced internalization of LPA_1_ and transferrin receptors via a clathrin- and cholesterol-dependent manner (Urs et al., 2005); LPA stimulated micropinocytosis via Rho GTPases and subsequent actin modification in *Entamoeba histolytica*, an anaerobic parasitic amoebozoan (Apte et al., 2022).

Outside VE cells, *Enpp2* mRNA is highly expressed in the choroid plexus, ciliary epithelium, and organizing centers in the developing neural nervous system, including the midbrain–hindbrain boundary and floor plate (Koike et al., 2006; Bächner et al., 1999; Koike et al., 2011). Because these cells secrete cerebrospinal fluid, aqueous humor, morphogens, and axon guidance molecules, autotaxin may play a role in promoting secretion in general, as we showed in yolk sac VE cells.

Furthermore, increased *Enpp2* expression is associated with many types of cancers, such as melanoma, breast cancer, glioblastoma, lung adenocarcinoma, and ovarian cancer (Leblanc and Peyruchaud, 2015; Peyruchaud et al., 2019). Previous studies have shown that cancer cells alter endocytosis and subsequent intracellular trafficking to favor their survival and proliferation by changing cell signaling, cell fate determination, directed cell movement, and cytokine secretion (Mellman and Yarden, 2013). In addition, *Enpp2* was identified as one of 90 genes associated with drug resistance in cancer (Tang et al., 2020). Therefore, elucidation of the roles of autotaxin in membrane trafficking may lead to the development of new drugs for cancer treatment in addition to the current development of autotaxin inhibitors in cancer therapy.

## MATERIALS AND METHODS

### Antibodies

The primary antibodies used were obtained from the following companies: anti-alpha-fetoprotein Ab-2 (1:100 for immunohistochemistry, 1:1000 for Western blotting; RB-365) from Thermo Fisher Scientific, anti-EGF receptor (1:1000, 610017) from BD Biosciences, anti-actin (1:10000, A2066) and anti-phospho-ERK1/2 (1:1000, 05-797R) from Sigma-Aldrich, anti-ERK1/2 (1:1000, 9102) from Cell Signaling Technology, anti-VEGF Ab-1 (1:1000, RB-222) from NeoMarkers, and anti-PECAM-1 (1:250, sc-1506) from Santa Cruz Biotechnology. The secondary antibodies used were Cy3-conjugated goat anti-rabbit IgG (1:500, 111-165-003) from Jackson ImmunoResearch, Alexa Fluor 633–conjugated donkey anti-goat IgG (1:1000, A21082) from Invitrogen, Cy3-labeled goat anti-mouse IgG (1:500, 115-165-003) from Jackson ImmunoResearch, HRP-conjugated goat anti-mouse IgG (1:1000, AP124P) from Millipore, HRP-conjugated goat anti-rabbit IgG (1:1000, 111-035-144) from Jackson ImmunoResearch, and HRP-conjugated goat anti-rat IgG (1:1000) from Chemicon.

### Animal experiments

*Enpp2*-knockout mice were generated and maintained as previously described (Koike et al., 2009). C57BL/6-green mice, C57BL/6-Tg(CAG-EGFP)C14-Y01-FM131Osb (developed by Masaru Okabe, Osaka University) were obtained from the BioResource Center, RIKEN Tsukuba Research Institute. All the experiments using animals were approved by the Animal Care and Use Committee of the University of Tsukuba and performed under its guidelines. Noon of the day on which a vaginal plug was observed was taken as embryonic day 0.5 (E0.5).

### *Ex vivo* whole-embryo culture

Whole-embryo culture was performed as described previously (Koike et al., 2009). In brief, embryos were dissected at E7.5 and cultured in 100% rat serum (Charles River) supplemented with 2 mg/ml glucose in a culture bottle placed in a rotation drum culture system (Ikemoto Rika) at 37°C under 5% O_2_, 5% CO_2_, and 90% N_2_ for 24 h. In the pharmacologic experiments, embryos were incubated with the following reagents: 3-(4-[4-([1-(2-chlorophenyl)ethoxy]carbonyl amino)-3-methyl-5-isoxazolyl]benzylsulfanyl) propanoic acid (Ki16425, Sigma; 10 µM), (*R*)-phosphoric acid mono-[2-amino-2-(3-octyl-phenylcarbamoyl)-ethyl] ester (VPC23019, Avanti Polar Lipid; 10 µM), and (*S*)-(+)-2-methyl-1-[(4-methyl-5-isoquinolynyl)sulfonyl] homopiperazine dihydrochloride (H1152, Calbiochem; 0.1 mM), phenylarsine oxide (Sigma-Aldrich; 0.1 µM), monensin (Nacalai Tesque; 50 nM), leupeptin (Peptide Institute; 25 µg/ml), 5-(*N*-ethyl-*N*- isopropyl)-amiloride (EIPA, MedChemExpress; 50 µM), dynasore (Tocris Bioscience; 20 µM), and pitstop 2 (Selleck Chemicals, 10 µM).

### RNA isolation and RT-PCR

Total RNA was isolated from yolk sacs of E8.5 mice by use of Sepasol I (Nacalai Tesque). Reverse transcription was performed by use of oligo(dT)_12–18_ and Superscript II reverse transcriptase (Invitrogen). Single-stranded cDNA was amplified by means of PCR using AmpliTaq Gold (Applied Biosystems) with the following primers:

*Actb*: 5′-TGCTGTCCCTGTATGCCTCTG-3′ (Forward, F) and

5′-TGATGTCACGCACGATTTCC-3′ (Reverse, R);

*Egfr*: 5′-TTAATCCCGGAGAGCCAGAC-3′ (F) and

5′-AGGCTCAGAAAGTGGTCTTC-3′ (R);

*Tgfb1*: 5′- GTGCTCGCTTTGTACAACAG-3′ (F) and

5′- ATCCCGTTGATTTCCACGTG-3′ (R);

*Vegfa*: 5′- TTTCTGCTCTCTTGGGTGCA-3′ (F) and

5′- TCTGAACAAGGCTCACAGTG-3′ (R).

Reaction products were electrophoresed on a 1% agarose gel containing ethidium bromide and visualized by use of the FAS III system (Toyobo). Real-time RT-PCR of *Egfr and Actb* was performed by use of the CFX Connect Real-Time PCR System (Bio-Rad) and SsoAdvanced Universal SYBR Green Supermix (Bio-Rad). For each sample, the relative amount of mRNA was determined by normalization to the level of the *Actb* gene.

### SDS-PAGE and immunoblot analysis

Protein was extracted from isolated yolk sacs in a lysis buffer (50 mM Tris-HCl pH7.5, 150 mM NaCl, 0.1% Triton X-100) containing Complete Protease Inhibitor cocktail (Roche). After centrifugation at 4°C for 15 min at 20,000×***g***, the concentration of the supernatants was measured by use of a Micro BCA Protein Assay kit (Pierce). Proteins were denatured in a sample buffer for 5 min at 95°C, resolved by means of SDS-PAGE, and transferred onto an Immobilon-P PVDF membrane (Millipore). The membranes were blocked in 5% skim milk (Nakalai Tesque) in PBS containing 0.1% Tween 20 (Sigma-Aldrich) for 1 h and incubated with primary antibodies overnight at 4°C. The next day, the membranes were incubated with HRP-conjugated secondary antibodies at room temperature for 1 h. Signals were detected by use of ECL Western Blotting Detection Reagent (Amersham) according to the manufacturer's instructions.

### Immunohistochemistry

For whole-mount immunohistochemistry of AFP protein on the surface of the yolk sac, yolk sacs were isolated from E8.5 embryos and incubated with an anti-AFP antibody (1:100) in PBS for 3 h. After being washed with PBS, yolk sacs were fixed with 4% paraformaldehyde (PFA) in PBS for 10 min and embedded into an OCT compound (Sakura Finetek Japan). Ten-micrometer-thick slices were made by use of a cryostat CM1850 (Leica) and collected on MAS-coated glass slides (Matsunami). They were then incubated with Cy3-labeled anti-mouse IgG (1:500, Jackson ImmunoResearch) and observed by use of a confocal microscope (LSM510, Zeiss).

### Collection of extraembryonic coelomic fluid (ECF)

ECF was aspirated by use of a fine-tip glass needle. About 1 µl of the ECF was obtained from 1 E8.5 embryo. The ECF collected from three to five mouse embryos was used for Western blot analysis or ELISA assay.

### Measurement of growth factors

Amounts of VEGF-A and TGF-β1 in the ECF and yolk sac were determined by use of a mouse VEGF Quantikine Colorimetric Sandwich ELISA kit (R&D Systems) and a TGF-β1 E_MAX_ ImmunoAssay System (Promega), respectively.

### Endocytosis assay of dextran, EGF, and transferrin

Whole embryos with intact yolk sacs dissected at E8.5 were incubated with 20 µg/ml Alexa Fluor 488-conjugated transferrin (Molecular Probes) or 100 µg/ml dextran rhodamine B 70,000 MW (Molecular Probes) in Dulbecco's modified Eagle's medium at 37°C for 3 min. After being washed, whole embryos were mounted on a glass-bottomed dish (Matsunami glass), and the yolk sac was observed by use of a laser scanning confocal microscope (LSM510, Zeiss) at room temperature. For quantitation of fluorescence intensity, the mean intensity in endosomes in each image was measured by use of Image J software (https://imagej.nih.gov/ij/).

### Quantification of HRP uptake

For HRP uptake assay, embryos were incubated with 100 µg/ml HRP (Sigma-Aldrich) at 37°C for 3 min. After being washed, the yolk sacs of individual embryos were separately removed and lysed in a lysis solution. HRP activity in the yolk sac was determined by measurement of the OD at 450 nm after incubation with 3,3′,5,5′-tetramethylbenzidine (Sigma-Aldrich) at 37°C for 10 min. The amounts of HRP were determined on the basis of the calibration curves drawn using the standard HRP protein and were normalized on the basis of the protein concentration determined by use of a Micro BCA kit (Pierce). Genotypes were determined by use of the genomic DNAs isolated from each embryo.

### Tetraploid complementation experiment

Tetraploid/diploid chimeric mice were made from diploid *Enpp2*^−/−^ embryos and tetraploid WT embryos essentially as reported previously (Shimizukawa et al., 2005; Tanaka et al., 2009). To mark tetraploid cells, they were produced from EGFP-expressing C57BL/6 mice (Shimizukawa et al., 2005). Electrofusion of blastomeres was performed at the two-cell stage by means of an ET-3 embryonic cell fusion system (Fujihira industry) according to the manufacturer's instructions. *Enpp2*^−/−^ embryos were generated by *in vitro* fertilization of *Enpp2* heterozygous mice. Diploid embryos at the eight-cell stage and tetraploid embryos at the four-cell stage were aggregated and transferred to the uteri of pseudopregnant recipient mice at 2.5 days postcoitus. Embryos were collected at E9.5, observed, and photographed by use of an epifluorescence stereomicroscope (MZ FLIII, Leica). After the chimeric embryos with EGFP-positive yolk sacs and EGFP-negative embryos proper were collected, DNAs were extracted from the embryos proper and genotyped.

### Statistical analysis

Statistical analysis was performed with KaleidaGraph (Synergy Software) and Origin 2022 (OriginLab). Data were expressed as means±SEMs. Statistical significance was determined by means of one-way analysis of variance (ANOVA) and the *t*-test. Probability values less than 0.05 were considered significant.

## Supplementary Material

10.1242/biolopen.060081_sup1 Supplementary information
