# Supplementary material for: The autotaxin-LPA axis promotes membrane trafficking and secretion in yolk sac visceral endoderm cells
Source: Biol Open. 2023 Oct 30;12(11):bio060081. doi: 10.1242/bio.060081 (PMC10629499; doi:10.1242/bio.060081)
Supplement: Supplementary information [file biolopen-12-060081-s1.pdf]

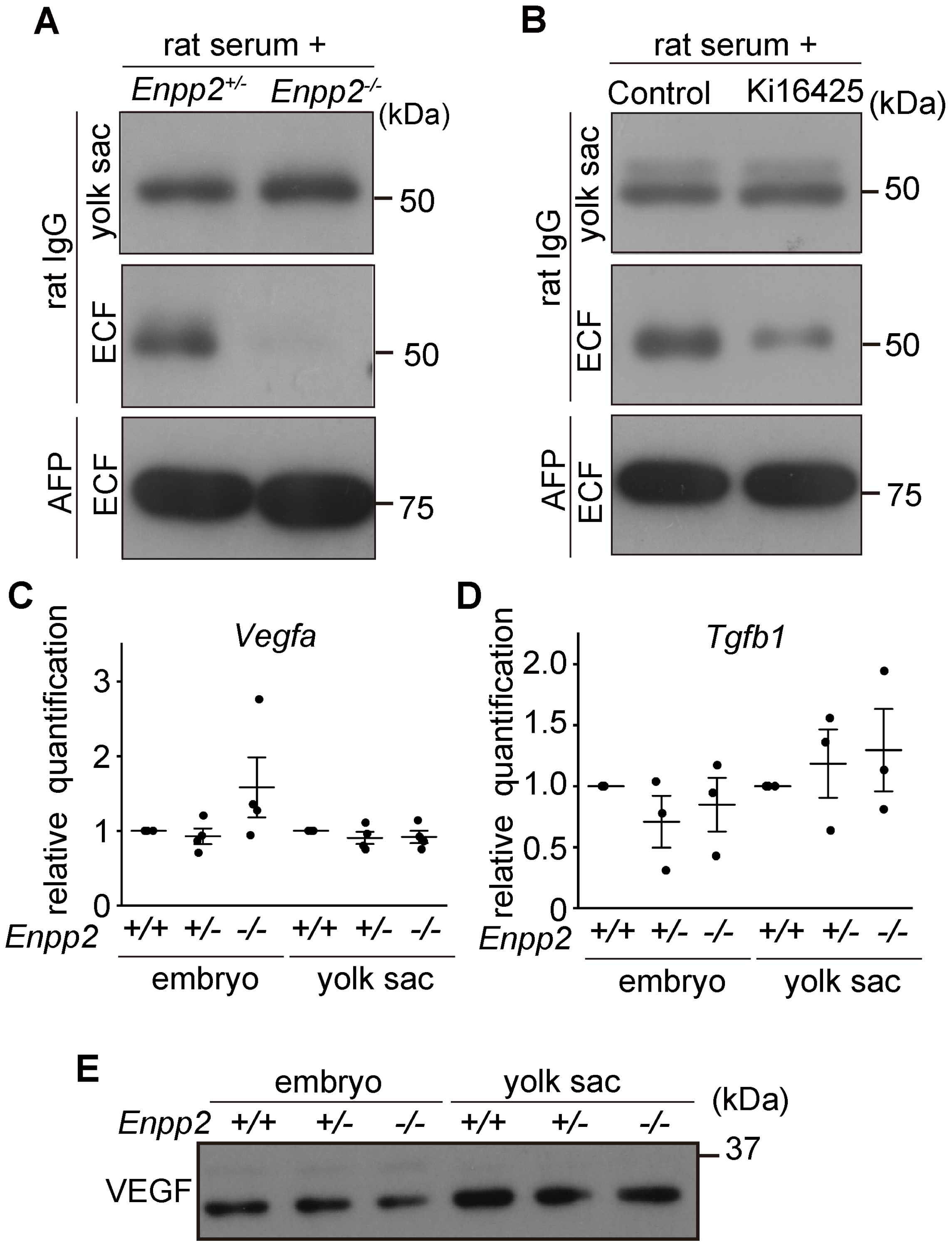

Biology Open • Supplementary information

Fig. S1. Supporting data related to other figures

(A) Another experiment of transcytosis of rat IgG across the VE cells (related to Fig. 1D). The amount of rat IgG in yolk sac cells was not changed, but IgG in the ECF was lower in the *Enpp2*$^{-/-}$ embryos than in the control. AFP protein was used as the internal control. (B) Another experiment of effects of LPAR on transcytosis of rat IgG across the VE cells (related to Fig. 1F). The amount of rat IgG in yolk sac cells was not changed, but IgG in the ECF was lower in the embryos treated with 10 μM Ki16425 for 1 d than in the control. AFP protein was used as the internal control. (C) The relative mRNA expression level of *Vegfa* and *Tgfb1* in the embryo and yolk sac of *Enpp2*$^{+/+}$, *Enpp2*$^{+/-}$, and *Enpp2*$^{-/-}$ at E8.5 were quantified (related with Fig. 4E, n = 3). (E) Another example of Western blot analysis of VEGF in the embryo and yolk sac of E8.5 mice (related with Fig. 4F).

Biology Open • Supplementary information

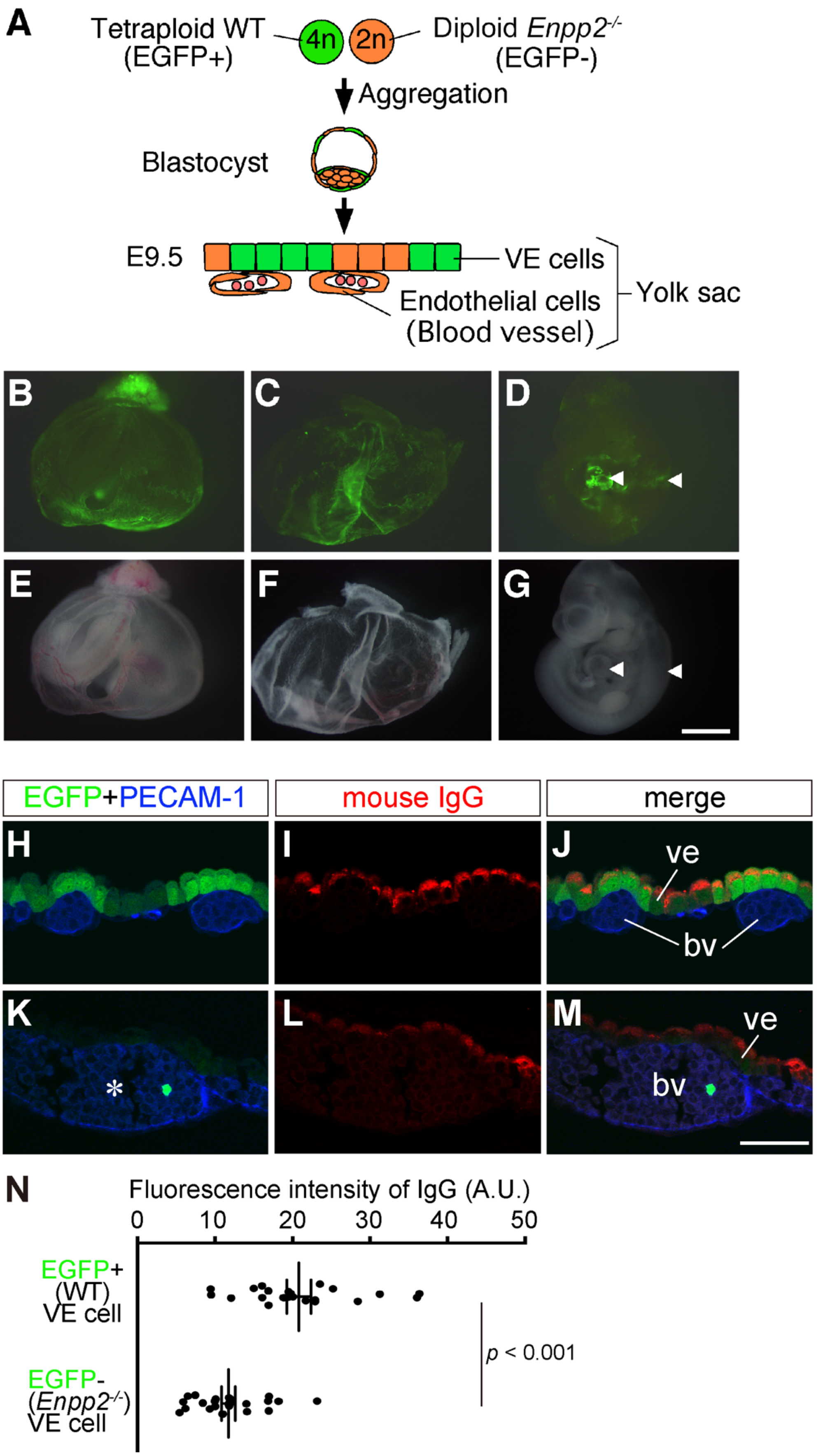

Biology Open • Supplementary information

Fig. S2. Tetraploid complementation experiment

(A) Strategy for the tetraploid complementation experiment. Tetraploid WT embryos expressing EGFP were aggregated with diploid *Enpp2*$^{-/-}$ embryos (EGFP-negative) and transferred to the uteri of pseudopregnant mice. Tetraploid cells contribute to extraembryonic tissues, but not to the embryos proper, whereas diploid *Enpp2*$^{-/-}$ cells can contribute to both embryonic and extraembryonic tissues. (B-G) Whole-mount images of an E9.5 chimeric mouse embryo. EGFP fluorescence (B-D) and appearance (E-G) of the whole embryo (B,E), yolk sac (C,F), and embryo proper (D,G) are shown. The arrowheads in (D,G) indicate EGFP-positive cells in the heart and neural crest cells of the embryo. (H-M) Section of the yolk sac of the chimeric embryo shown in (B-G). Immunostaining for PECAM (blue) and mouse IgG (red) is shown. An area containing many EGFP-positive cells (H-J) and an area lacking EGFP-positive cells (K-M) are shown. The asterisk in (K) indicates dilated blood vessels. Abbreviations: bv, blood vessel; ve, visceral endoderm. Scale bars: 1 mm in (B-G) and 50 µm in (H-M). (N) Quantification of endocytosed IgG in VE cells. The levels of the fluorescence intensity of mouse IgG in EGFP-positive (n = 22) and EGFP-negative (n = 25) VE cells are shown (unpaired t-test).
